# Altered cortical network in female with adolescent idiopathic scoliosis

**DOI:** 10.1186/1748-7161-10-S1-O46

**Published:** 2015-01-19

**Authors:** Winnie CW Chu, Lin Shi, Defeng Wang, Jack CY Cheng

**Affiliations:** 1 Department of Imaging and Interventional Radiology, The Chinese University of Hong Kong, Hong Kong; 2 Department of Medicine and Therapeutics, The Chinese University of Hong Kong, Hong Kong; 3 Department of Orthopaedics and Traumatology, The Chinese University of Hong Kong, Hong Kong

## Objective

The aim of the study was to investigate abnormalities in the organization of the brain cortical network in patients with AIS based on MR data. Adolescent idiopathic scoliosis (AIS) is a three dimensional spinal deformity that affects teenagers especially girls between the ages of 10-16 at prevalence of 1-4%. The etiopathogenesis remains uncertain but scientific studies have suggested it is a multifactorial disease included different intrinsic and extrinsic factors. A recent study has demonstrated the abnormal cortical thickness between patients with AIS and healthy controls. Cortical thickness is strongly correlated between regions that are axonally connected. Hence, the hypothesis of altered organization pattern of large-scale structural network in patients with AIS has been tested in this study.

## Materials and methods

This study included 42 girls with severe idiopathic scoliosis (14.7+/-1.3 years old, range 13-18) and 41 age-matched normal controls (14.6+/-1.4 years old, range 12-18). The brain cortexes were partitioned into 154 cortical regions based on a spherical atlas. The alteration of interregional correlation, small-world efficiency, hub distribution, and regional nodal characteristics were tested and analyzed by cortical thickness measurement, interregional correlation, and graph theory.

## Results

The results demonstrated that the cortical network of AIS fully preserved the small-world architecture and organization, and further verified the hemispheric asymmetry of AIS brain. Increased central role of temporal and occipital cortex, decreased central role of limbic cortex, increased connectivity in different cortical regions and decreased structural connectivity between hemispheres were observed in AIS.

## Conclusions

patients with AIS preserved the small-world efficiency but experienced altered interregional correlation, hub distribution and nodal characteristics. They were associated with changed between-hemisphere connectivity, and ability of communication of cortical networks. Results were consistent and comparable to our previous neuroanatomical findings, and they could enhance the understanding of neurological changes in AIS.

## Interregional correlation

The interregional correlation matrix of a group is generated by calculating the correlation coefficients across subjects between the thicknesses of each pair of cortical regions.

## Small-world efficiency

The network efficiency metrics are used to refect the efficiency in communication and organization over a network system.

## Hub distribution

Hub distribution means the cerebral distribution of the brain regions identified as hubs.

## Regional nodal characteristics

The regional nodal characteristics are referring to the metrics used to describe the network characteristic of each node (not the whole network), including the regional efficiency, betweenness centrality, et al.

